# An Improved Sensing Method of a Robotic Ultrasound System for Real-Time Force and Angle Calibration

**DOI:** 10.3390/s21092927

**Published:** 2021-04-22

**Authors:** Kuan-Ju Wang, Chieh-Hsiao Chen, Jia-Jin (Jason) Chen, Wei-Siang Ciou, Cheng-Bin Xu, Yi-Chun Du

**Affiliations:** 1 Department of Biomedical Engineering, National Cheng Kung University, No.1, University Road, Tainan 70101, Taiwan; christine@brainnavi.com (K.-J.W.); chenjj@mail.ncku.edu.tw (J.-J.C.); p86104152@gs.ncku.edu.tw (C.-B.X.); 2 Brain Navi Biotechnology Co., Ltd., No.66-1, Shengyi 5th Rd. Zhubei City, Hsinchu County 302041, Taiwan; jerrychen@brainnavi.com (C.-H.C.); weisiang@brainnavi.com (W.-S.C.); 3 China Medical University Beigang Hospital, No.123, Xinde Road, Xinjia Village, Beigang Township, Yunlin County 65152, Taiwan; 4 Medical Device Innovation Center, National Cheng Kung University, No.1, University Road, Tainan 70101, Taiwan

**Keywords:** robotic ultrasound system (RUS), multichannel pressure sensors, inertial measurement unit (IMU), low cost, phantom test

## Abstract

An ultrasonic examination is a clinically universal and safe examination method, and with the development of telemedicine and precision medicine, the robotic ultrasound system (RUS) integrated with a robotic arm and ultrasound imaging system receives increasing attention. As the RUS requires precision and reproducibility, it is important to monitor the real-time calibration of the RUS during examination, especially the angle of the probe for image detection and its force on the surface. Additionally, to speed up the integration of the RUS and the current medical ultrasound system (US), the current RUSs mostly use a self-designed fixture to connect the probe to the arm. If the fixture has inconsistencies, it may cause an operating error. In order to improve its resilience, this study proposed an improved sensing method for real-time force and angle calibration. Based on multichannel pressure sensors, an inertial measurement unit (IMU), and a novel sensing structure, the ultrasonic probe and robotic arm could be simply and rapidly combined, which rendered real-time force and angle calibration at a low cost. The experimental results show that the average success rate of the downforce position identification achieved was 88.2%. The phantom experiment indicated that the method could assist the RUS in the real-time calibration of both force and angle during an examination.

## 1. Introduction

The ultrasound imaging system is a universal and common clinical detection method in preoperative examinations, and is characterized by real-time imaging, noninvasiveness, non-ionizing radiation, and lower cost; therefore, this medical imaging equipment is extensively used in medical institutions. However, while the ultrasound imaging system has a very high clinical usage rate, it relies heavily on doctors’ operating experience. Moreover, repeated applications of force at specific angles are required in the detection process to obtain clear ultrasonic images [1]. During the probe scanning process of conventional ultrasonic detection, the doctor has to maintain a grip on the probe with constant force and specific angle for a long time. Thus, the hand is likely to shake, which results in unsharp images, and is likely to cause occupational injury in the long term, e.g., shoulder pain [2]. At present, an integrated application based on a robotic ultrasound system (RUS) is suitable for improving the above issues. The robot system has apparent advantages (stability, high success rate, repeatability, dexterity, and operability). Thus, the performance of the RUS in image capture and control could be effectively improved, which increases the efficiency of ultrasonic image detection [3,4]. In addition, in recent years different robot arms integrated with real-time ultrasonic images were extensively used for preoperative and intraoperative examinations by different departments, and because the robot arms have high stability and system reproducibility, human errors have been greatly reduced [5,6]. According to an investigation by BIS Research in 2018, the global market scale of medical robot arms was USD 5.08 billion in 2017, and has been estimated to reach USD 12.6 billion by 2025, for a compound growth rate of 12.0%, and there would be more companies devoted to the field to perform related technological research and development [7]. With the popularization of surgical robots, the robotic application developments of different departments were accelerated, including rehabilitation, minimally invasive surgery, medical image navigation systems, etc. [8,9,10]. However, ultrasound imaging equipment are very common examination instruments in health facilities. To import a robotic ultrasound examination with efficiency and low cost, the current RUSs were mostly integrated with the ultrasound imaging equipment that already exist in the health facilities by using a self-designed fixture to connect the probe to the arm.

In terms of ultrasonic applications, many research teams have proposed integrating ultrasound with robots for diagnosis and validated the flexibility and success rate. The results proved that the robot arm could proficiently and repeatedly execute the action command set by the user, and it has considerable potential in various surgical operations. Related studies have indicated that medical robot arms would be an indispensable part of clinical medicine in the near future [11]. In 2013, Mustafa et al. proposed an automatic scanning RUS for the liver, which featured an integrated camera, and could be inserted through the skin surface of the navel or nipple to analyze and scan the optimum location of the liver and conduct image analysis, and the experimental results showed that the precision of the system was 94% [12]. However, the research applied force to the ultrasound probe by using Constant Applied Force Control. When the image was abnormal, the medical care personnel needed to adjust the force and angle. There were neither real-time force feedback nor error correction mechanisms. In 2019, Huang et al. proposed a robot-assisted and remote-control ultrasonic scanning system for three-dimensional imaging where the operator controlled the six-axis robot arm remotely to drive the ultrasonic probe to scan the skin surface. Based on the internet and four cameras, the operator could instantly observe the robot arm and patient, and appropriately adjust the motion mode of the probe. The quantitative experimental result showed that the error of the volume measurement was less than 1.1% [13]. This research also revealed that, in future examinations, a lot of fine-tunings will be required during the scanning because the skin surfaces of the human body are usually complicated. In addition, users need to focus on the scanning pathways in the image and the moving probe. Without the information of force feedback, it is hard for the medical care personnel to make the correct judgment [13]. On the other hand, in a literature review of calibration method applications, Chen et al. proposed a set of ultrasound-guided needle insertion robotic system for percutaneous puncture in 2021. In the system calibration, this research used the coordinate system calibration method to calibrate the ultrasonic system and robotic arm. To begin with, the calibration device consisted of a 3-DOF moving mechanism, water tank, and multiple groups of nylon wires. Then the moving mechanism was designed to allow the puncture robot to move around the water tank and keep the US probe perpendicular to the water surface. In the water tank, three groups of nylon wires were fixed in the designated position and oriented parallel to the water surface. Finally, according to the distance difference between the coordinate system of the fixed position of nylon wire and the coordinate system of the needle line, the estimation was conducted, and the calibration was completed. This method could effectively correct the image by fixing the position of the nylon wire, but neither angle error nor self-correction of ultrasonic probe on the robot arm was discussed [14]. In fact, the self-correction of the system could be seen as a resilient problem. In the related studies, this problem was explained in” Toward a resilient manufacturing system” brought up by Zhang and Van Luttervelt in 2011. In this paper, the concept of engineering resilience was revisited and clarified [15]. We referred to the remarks and multiple cases in the research of Zhang and Van Luttervelt to optimize a RUS, especially the system correction. 

The above studies show that the RUS has high stability and reproducibility in ultrasonic examinations and could greatly reduce human errors and potential occupational injuries. Moreover, related studies show that, as the epidemic situation of COVID-19 gets worse, a medical robot arm could assist in specimen collecting [16,17,18,19]. However, even after development in recent years, the robot arm is still unable to fully replace clinical professionals due to the absence of a haptic forced feedback mechanism. Thus, the force at specific angles for conventional ultrasonic examination could not be effectively quantized [20,21,22]. The past relevant studies have shown that the development and application of pressure sensors for robots were quite extensive. Take machine tactile for example, McInroe et al. proposed a novel controllable stiffness tactile device that incorporated both optical sensing and pneumatic actuation in 2018. The changes of the traces of the trajectories from LED were extracted through the LED and camera in the pressure capsule, then did the image analysis to get the direction of the thrust and force value. This architecture has a very high potential for development of future realization of the robot’s fingertip tactile sensation [23]. However, the study mentioned in future work that the size could be minimized to the size of fingertips, but the final size was restricted by the size of the internal camera. In fact, with a camera of Principles of Optics, although the surface displacement and force data could be converted into a three-dimensional image to achieve tactile visualization through the Tessellation point on the plane caused by the force, its structure was restricted by the lens specifications and size, which made it difficult for its integration and application of robotic arms [24,25,26]. In addition, as the commercially available collaborative robot arm grips the ultrasonic probe with a gripper, different fixture and probe models cannot be effectively fitted, and the ultrasonic probe may shake slightly during the measurement process, which leads to errors in measurement angles. Therefore, this study proposed an improved sensing method of the RUS, for real-time ultrasonic probe force and angle monitoring, which could easily and rapidly combine the probe and arm. By applying a multichannel force-sensing technique and novel sensing structure design, the magnitude and direction of the force of the probe to the body surface could be accurately known at a low cost. Meanwhile, the information could be instantly returned to the system for analysis. The analysis result could be synchronously transferred to the robot arms for angle compensation, which was quite important for high Degree of Freedom (DOF) robot arms in real-time ultrasonic robot manipulation. Furthermore, according to our knowledge, present studies mostly use a self-designed fixture for connecting the probe to the robot arm; however, as the specifications and sizes of the probes are different during integration, inconsistencies often occur in the angle correction, which lead to false ultrasonic scanning angle estimations. As the angular error of integration increases, this situation may result in mechanism instability during long duration operations. The multichannel inertial sensing technique was used in this study, meaning the angular error between the probe and arm could be instantly identified, and immediately fed back to the system side. In order to increase system accuracy and reduce the difficulty level of the system integration, this system was designed based on a six-axis robot arm. After the scan path was set up by the clinician, the system could perform ultrasonic scanning based on the usage scenarios of different departments, where the parameters were corrected according to the inertial measurement unit (IMU) and real-time forced feedback image results to obtain high-quality ultrasonic images. 

## 2. Materials and Methods

### 2.1. Architecture of the System

**Figure 1:**
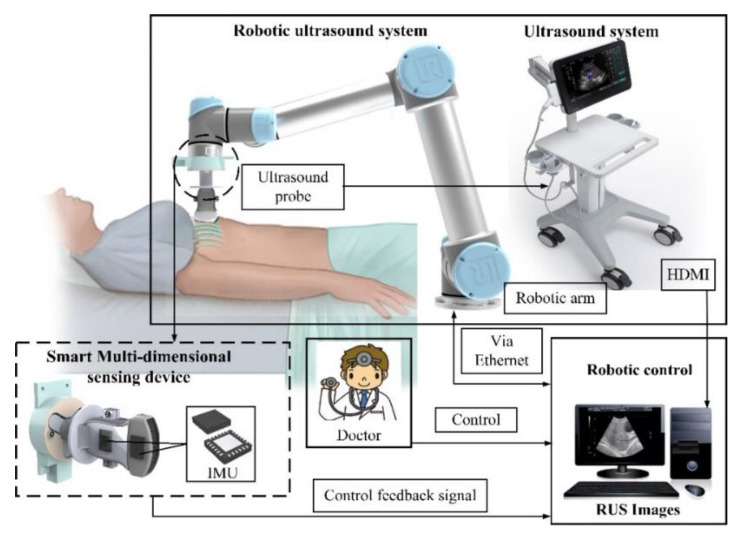
System architecture.

The architecture of this system was comprised of three items, the six-axis robot arm system, an ultrasound imaging system, and an adjustable fixture mechanism design, as shown in Figure 1. As the six-axis robot arm used in this study was driven by six motors in different positions, each motor could provide uniaxial rotary motions. In terms of the DOF, the six-axis robot arm satisfied the six DOF in a 3D space, which was sufficient for conventional ultrasonic scanning. The ultrasound imaging system used the T-3300 for scanning (T-3300, BenQ Medical, Taipei city, Taiwan) developed by BenQ Medical. In addition to a sharp image quality, through integration with a robot arm, the system featured high mobility and digital intelligent management. Integrated with a touch screen, the supports intelligentized the gesture operation for lightness, flexibility, and fast booting. It was also highly flexible in an emergency and appropriate for serious symptom examinations for home care. Finally, in order to maintain a stable movement of the ultrasonic probe and force at a specific angle, the adjustable fixture mechanism was integrated with thin film pressure sensor and IMU, meaning the errors were compared for the angle of the ultrasonic probe and the applied force was measured instantly in the automatic scanning process. When the ultrasonic probe shaked during scanning, it led to angle errors. Thus, when the force applied by the robot arm was insufficient, feedback of real-time data analysis was provided, and the robot arm performed real-time angle or force compensation to avoid the preset angle being nonsynchronous with the actual measurement angle. With this architecture, the system could upgrade the quality of the ultrasonic images. To make the different robot arm models compatible with the ultrasonic probe, the system uses an adjustable fixture mechanism, VGA, and an HDMI image capture card to capture ultrasonic images per second. In the analysis process, the motion of the robot arms, the image obtained in the scanning process, and the angle information were transferred to the back-end analytic system synchronously, which provides medical care personnel with a reference for each examination. The mechanisms were detailed as follows. 

### 2.2. Multichannel Force Sensing

**Figure 2:**
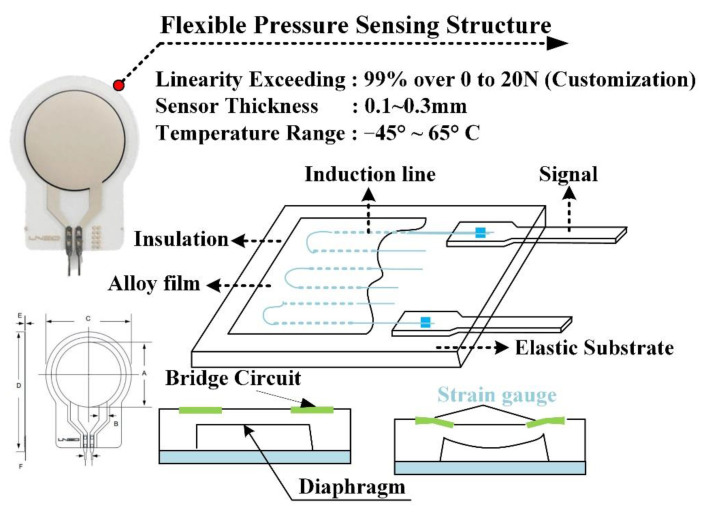
Structural representation of the flexible thin film pressure sensor.

The pressure sensor used in this study was GD10-20N developed by Uneo Inc. The sensor was fabricated using a piezoresistive polymer composite and screen printing as shown in Figure 2. Prior studies have described the typical process method for force-sensing film [27]. First, the electrode pattern and signal transmission line were printed on the substrate. In the course of production, the signal line was wrapped in the insulating layer to avoid signal transmission interference. As GD10-20N was produced using an imprinting and Flexible Printed Circuit (FPC) technique, the line spacing in the insulating layer was 0.1 mm. Thus, a precise circuit could be designed in a micro shape, and fabrication could be customized according to client-side requirements. GD10-20N, which was characterized by lightness, flexibility, and compactness, has good environment fitness, and remains sensitive at harsh ambient temperatures (−40°~65 °C). The output of sensors could be adjusted by an amplifier circuit to provide higher voltage range and the resolution. It could increase the correlation between the output and the Newton force (0 N~20 N), which achieved the linearity of regressive analysis to 99%. The sensors could be reused ten million times. According to prior studies, the thin film pressure sensor has the advantages of system integration, as well as data linearity and reliability [28,29,30]. 

### 2.3. Architectural Design of the Bilateral IMU

**Figure 3:**
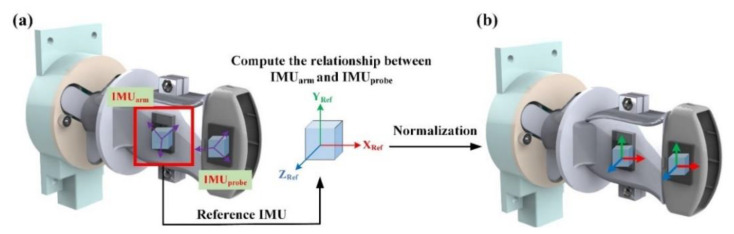
Schematic diagrams before and after IMU Euler angle correction: (**a**) schematic diagram of the Euler angle orientation of the IMU fixed to the robot arm; (**b**) schematic diagram of the Euler angle orientation of the IMU mapped on the ultrasonic probe after correction.

In the movement of the robot arm, the ultrasonic probe easily produced angular errors from the excessive vibration. Once the angle of the ultrasonic probe was different from that of the robot arm, the scanned ultrasonic image result could cause errors in subsequent interpretations by the clinician. The bilateral IMU sensing architecture was used to analyze the angles from the ultrasonic probe and robot arm during scanning. The IMU used in this study was an MPU-6050 developed by TDK InvenSense, and comprised a three-axis gyroscope and a three-axis accelerometer. For precision tracking of both fast and slow motions, the parts featured a user-programmable gyro full-scale range of ±250, ±500, ±1000, and ±2000°/s (dps) and used 16-bit analog-to-digital converters (ADCs) for digitizing the gyroscope outputs. In this study, all of the operations were done under slow motion, so the minimal resolution could reach 0.1°. Moreover, the sensor had a built-in Digital Motion Processor (DMP), which could read data from the gyroscope and accelerometer. Afterwards, the motion processing algorithm was performed in the 200 Hz instruction cycle. Finally, the result was saved in the register for the user to access [31]. As the sensor could instantaneously detect variations, it was quite suitable for measuring minute angles in this study. When the robot arm gripped the ultrasonic probe for scanning, the adjustable mechanism and bilateral IMU inter-compares the angles to detect the consistency between the ultrasonic probe angle and the preset angle of the robot arm, and the IMU angle could be transformed according to the three-dimensional coordinates. The usage is shown in Figure 3. 

In order to ensure the consistency of the Euler angle orientation of the IMUs that were disposed on the robot arms (IMU_arm_) and ultrasonic probe (IMU_probe_) a correction formula was required for normalization. As the rotation parameters provided by the IMU were the Roll, Pitch, and Yaw of the Euler angle before the correction operation, the direction of the IMU [$\varphi_{a}\left(t\right)\rho_{a}\left(t\right)\theta_{a}\left(t\right)$] was calculated according to the three axial angular velocities [$\omega_{x},\;\omega_{y},\;\omega_{z}$] around the gyroscope, and the angular displacement could be obtained by performing integral calculation of the angular velocity [32].

(1) $$\{\begin{array}{c} \varphi_{g}\left(t\right)=\int_{t_{i}}^{t_{f}}\omega_{x}dt\\ \rho_{g}\;\left(t\right)=\int_{t_{i}}^{t_{f}}\omega_{y}dt\\ \theta_{g}\left(t\right)=\int_{t_{i}}^{t_{f}}\omega_{z}dt \end{array}$$

where the angle value and $\Delta_{t}$ value of the given (*t* − 1) approximate to

(2) $$\{\begin{array}{c} \varphi_{g}\left(t\right)=\varphi_{g}\left(t-1\right)+\omega_{x}\Delta_{t}\\ \rho_{g}\left(t\right)=\rho_{g}\left(t-1\right)+\omega_{y}\Delta_{t}\\ \theta_{g}\left(t\right)=\theta_{g}\left(t-1\right)+\omega_{z}\Delta_{t} \end{array}$$

Afterwards, the integral error of the gyroscope could be corrected using the measured value of the accelerometer, and the rotation matrix was obtained:

(3) $$R=\left[\begin{array}{ccc} r_{11} & r_{12} & r_{13}\\ r_{21} & r_{22} & r_{23}\\ r_{31} & r_{32} & r_{33} \end{array}\right]$$

where

(4) $$\{\begin{array}{c} r_{11}=\cos(\varphi )\cos(\rho )\\ r_{12}=-\sin(\varphi )\cos(\theta )+\cos(\varphi )\sin(\rho )\cos(\theta )\\ r_{13}=\sin(\varphi )\cos(\theta )+\cos(\varphi )\sin(\varphi )\cos(\theta )\\ r_{21}=\sin(\varphi )\cos(\rho )\\ r_{22}=\cos(\varphi )\cos(\theta )+\sin(\varphi )\sin(\rho )\sin(\theta )\\ r_{23}=-\cos(\varphi )\sin(\theta )+\sin(\varphi )\sin(\rho )\cos(\theta )\\ r_{31}=-\sin(\rho )\\ r_{32}=\cos(\rho )\sin(\theta )\\ r_{33}=\cos(\rho )\cos(\theta ) \end{array}$$

Moreover, if the ultrasonic probe was displaced, there would be errors in the bilateral IMU conversion angle, and this offset could be defined based on a trigonometric function variation. The IMU_arm_ could be regarded as the vertex of a reference and defined as follows [32]:

(5) $$x_{f}^{2}+y_{f}^{2}+z_{f}^{2}=P_{u}^{2}+P_{f}^{2}-2L_{u}L_{f}\cos\left(\gamma_{e}\right)$$

where the deviation angle $\left(\gamma_{e}\right)$ could be deduced to

(6) $$\gamma_{e}\left(t\right)=arcos\left(-\cos\left(\theta_{f}\left(t\right)-\theta_{u}\left(t\right)\right)\cos\left(\rho_{f}\left(t\right)\right)\cos\left(\rho_{u}\left(t\right)\right)-\sin\left(\rho_{f}\left(t\right)\right)\sin\left(\rho_{u}\left(t\right)\right)\right)$$

Thus, the directions of IMU_arm_ and IMU_probe_ were directly correlated. As the IMU_arm_ was fixed to the robot arms, IMU_arm_ was defined as the reference point. Afterwards, IMU_probe_ could perform coordinate coupling against IMU_arm_. First, the fixture must be parallel to the ultrasonic probe, and then the directions of IMU_arm_ and IMU_probe_ were recorded. The calculated rotation matrices of the displacement and deviation were $R_{u}^{-1}\left(0\right)$ and $R_{f}^{-1}\left(0\right)$ respectively. Finally, the correction rotation matrices [27] of IMU_arm_ and IMU_probe_ were defined as $R_{u}\left(t\right)R_{u}^{-1}\left(0\right)$ and $R_{f}\left(t\right)R_{f}^{-1}\left(0\right)$ respectively.

### 2.4. Novel Sensing Structure Design

**Figure 4:**
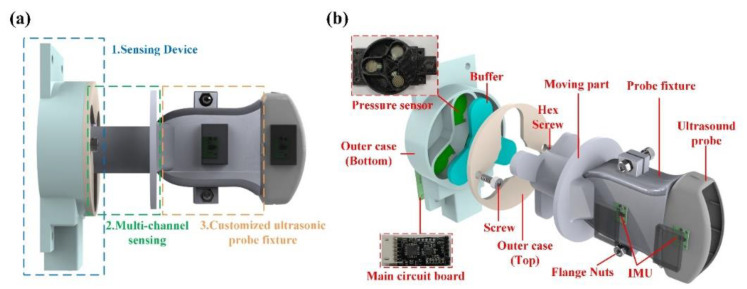
(**a**) Schematic diagram of the sensing structure; (**b**) schematic diagram of the internal structure.

In this study, the structure was preliminarily designed by 3D drawing software, implemented by 3D printing, and the overall volume size was 110 × 68 × 113 mm. The sensing structure in this study was designed by assembling multiple parts and comprised three major parts: (1) the sensing device; (2) the multichannel force-sensing structure; and (3) the adjustable structure of the ultrasonic probe fixture, as shown in Figure 4a. The sensing device included the main circuit, force sensing, the IMU, and power management, and its major functions included sensor data processing and Bluetooth wireless transmission. Moreover, the multichannel force-sensing structure was mobile and corresponded to three pressure sensors. When the structure deflects to a direction, the corresponding pressure sensor value was increased accordingly, and the direction of the force applied by the ultrasonic probe could be known from this data so as to provide medical care personnel with an operational reference. This adjustable ultrasonic probe fixture structure was provided for the most different ultrasonic probe models available on the market. The internal structure of the overall device is shown in Figure 4b. 

### 2.5. Hardware Design

**Figure 5:**
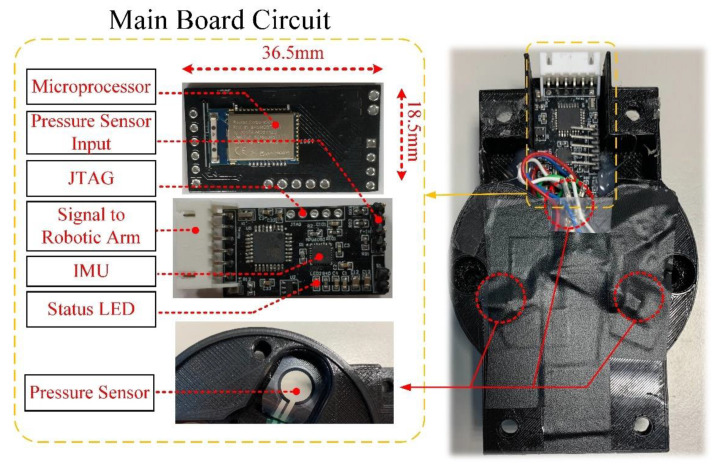
Stereogram of the hardware device.

In this study, the hardware design was fabricated by double-layer PCB. The top layer had a monolithic chip (nRF52832, Nordic Semiconductors, Norge) and a program access port. The bottom layer had the IMU sensing circuit, force-sensing reception, and power management. The overall size was 36.5 × 18.5 mm, as shown in Figure 5. As the nRF52832 monolithic chip used an ARM Cortex-M4F processor, 512 kB Flash, 12-bit A/D resolution, and built-in Bluetooth 5.0, it could efficiently execute the sensed data processing and low power consumption wireless transmission. Finally, for the calibration and system repeatability, we referred to the related literature and designed the experiments as below [33,34].

## 3. Experimental Design and Results

### 3.1. Multi-Point Diaphragm Force-Sensing Correlation Test 

**Figure 6:**
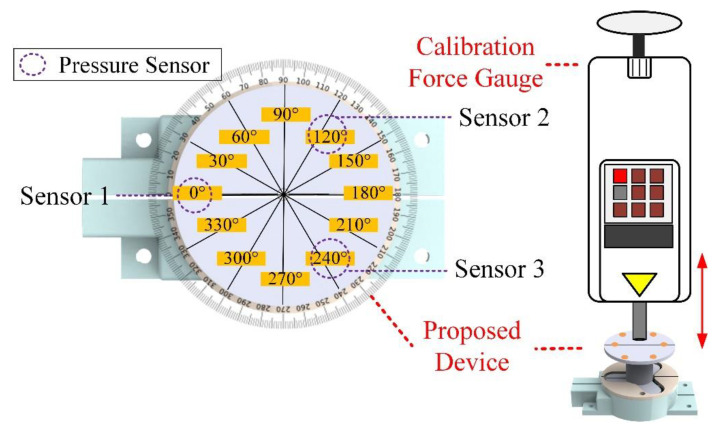
Schematic diagram of the multi-point diaphragm force-sensing correlation test.

A commercially available push pull gauge (HFG-HF-10, ALGOL, Taoyuan, Taiwan) for the multi-point diaphragm force-sensing correlation testing was used in this study. The instrument had ±0.2% full-scale (FS) precision, ±0.1% FS for replicated measurements, and the measurement range was 0 to 100 N, and resolution was 0.01 N. Additionally, the instrument could log the test data of 512 records and export the result, which was favorable for subsequent data analysis. The measurement errors induced by gravitation could be eliminated by the built-in self-correction module. Finally, the measurement result could be transferred through communication protocol RS-232 to the computer terminal and displayed. In this section, the commercially available thrust-tension meter applied force to the multi-point diaphragm force-sensing structure proposed in this study. The correlation and validity were validated according to the measured data so as to validate the success rate of the sensing points and guarantee mutual independence among the sensing points. The position where the pressure sensor was installed would apply 3 N, 5 N, 8 N, and 10 N of downforce as the testing force maintaining for over ten seconds, and the force was applied to each sensing point 100 times. Then the MCU performs A/D conversion, and the force was caculated by the force convertion equation and was saved for further analysis. The schematic diagram of the multi-point diaphragm force-sensing correlation test is shown in Figure 6. 

**Figure 7:**
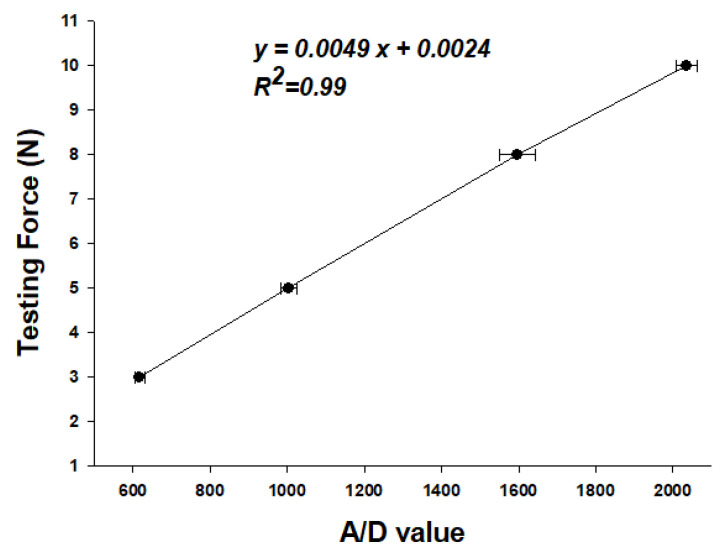
Linear regression results between the A/D values and testing force (N).

The experimental data presented in Table 1 show that different forces correspond to different A/D values, and the output force could be calculated by the regression curve equation. Figure 7 shows the results of the linear regressive analysis between the A/D values and testing force (*R*^2^ = 0.99), and Table 1 shows the errors of the output force that were calculated by the regression curve equation and the average error was below 4.2%. 

**Figure 8:**
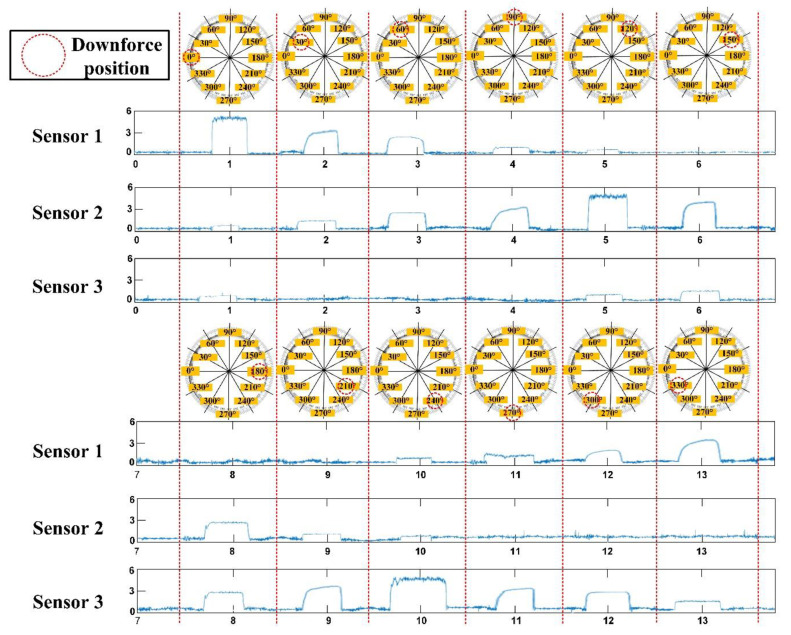
The different positions of the downforce angle and related force signals.

In order to verify that the system proposed in this study could identify the different positions of downforce based on three sensing points, we designed an experiment that 5 N were applied to each downforce position 100 times, and this experiment was repeated 10 times. Then, the measurements from the three sensors were used to compare the thresholds by the look-up table method to identify the position of the downforce. The angle of the downforce positions was 30° per movement, and the related signals are shown in Figure 8. All the data were transmitted in the wireless mode to the computer for analysis. The results show that the average success rate could reach 88.2% (Table 2). Moreover, we performed this experiment at different room temperatures (28 °C and 20 °C) to verify the influence of temperature. The results indicated that there were no statistically significant differences between the different temperature groups. 

### 3.2. Angle Correlation Validation of the Bilateral IMU

**Figure 9:**
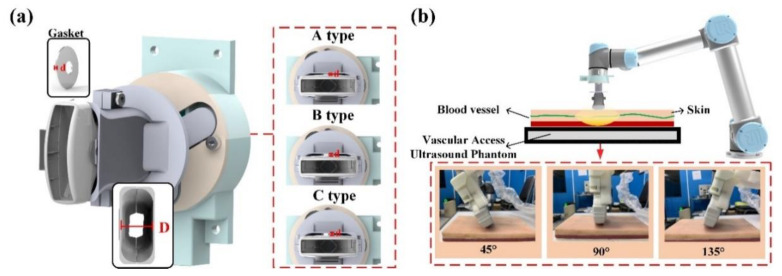
(**a**) Schematic diagram of the fixture structure molds with different clearances; (**b**) schematic diagram of the phantom test.

As the commercially available collaborative robot arm gripped the ultrasonic probe with a gripper, or used a special fixture for integration, the different fixture models and probes were unlikely to be effectively fitted. When the robot arm angle deviated from the ultrasonic probe angle, the measurement result would influence subsequent treatment; therefore, we used the structure design of a bilateral IMU to analyze the success rate of the linking angle between the robot arm and ultrasonic probe. In our prior study of IMU, a series of experiments were performed on the angular success rate of a single IMU to guarantee the success rate of the angular transformation [34,35,36]. To validate the system success rate, this study used a 3D printer (PING 300+, LINKIN FACTORY CO., LTD., Taiwan) applicable to multiple printing materials, including PLA, PETG, ABS, NYLON, TPE, and TPU to make the outer casing of the fixture. By adding different thicknesses of spacers in the experiment, we could obtain angular errors from the ultrasonic probe. In the experiment, the spacers in ascending order of thickness were Type A (*d* = 2.5 mm); Type B (*d* = 5 mm), and Type C (*d* = 10 mm), as shown in Figure 9a. When the experiment began, the fixture was disposed on the robot arm, and the robot arm performed ultrasonic phantom scanning on a 45°, 90°, and 135° fixed scan path. The scan at each angle, whose results were averaged, and errors were recorded, was performed ten times for over ten seconds each time, as shown in Figure 9b. 

**Figure 10:**
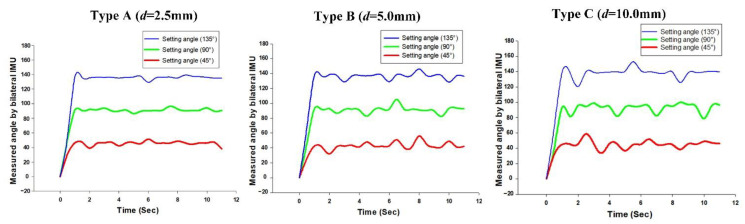
The variance of the measurement angle with different spacer thicknesses.

According to the experimental data in Table 3, the larger thickness of the spacer caused a greater error in the angles than that measured by the bilateral IMU (IMUarm and IMUprobe). In addition, the larger thickness would also cause the ultrasound probe to shake, which would make the IMUprobe deviate obviously from IMU arm in the horizontal plane, as shown in Figure 10. We set 2 degrees as a threshold for starting the calibration function to avoid the influence of variation during the RUS operation.

### 3.3. Phantom Test

**Figure 11:**
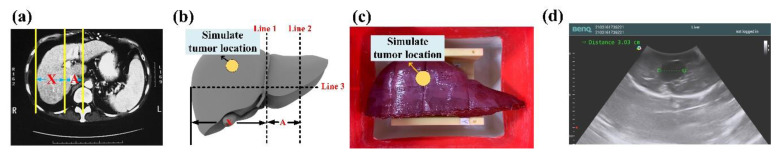
(**a**) Abdominal cavity phantom CT image; (**b**) 3D modeling by measuring method for liver on a CT image; (**c**) stereogram of the gel-prepared phantom; (**d**) phantom ultrasonic image and tumor location.

According to a literature review, the conventional ultrasonic examination (US) was the preferred method for preliminary sieving analysis of a hepatic tumor, as it could effectively display the location, size, form, internal echo, and blood flow condition inside the tumor. Thus, it had diagnostic value in diagnosing benign and malignant hepatic tumors [37,38]. To validate system accuracy, the self-made gel phantom and RUS were used for phantom testing. This gel phantom was fabricated using transparent silicone material (SI-040, RUIXIN ENTERPRISE CO., LTD., Taoyuan city, Taiwan) and 3D ABS or PLA printing molds, and the manufacturing process was improved by referring to the study of Ahmad et al. (2020) [39]. The related dimensions were molded according to the CT image; according to the CT image result in Figure 11a, Line 1 could be drawn in parallel with the right liver wall at the portal vein bifurcation, Line 2 was drawn in parallel with the innermost edge of the liver’s caudate lobe, and Line 3 was marked out between Line 1 and Line 2, which were normal to the portal vein and inferior vena cava, and extend to the right edge of the liver. The distance between Line 1 and Line 2 (i.e., Interval A) was the transverse diameter of the caudate lobe, while the distance along the right edge and Line 1 (i.e., interval X) along Line 3 was the transverse diameter of the right lobe hole sites of veins and the hepatic artery, as shown in Figure 11b. To meet the actual liver condition, the powder ratio could be adjusted in the phantom manufacturing process to make the phantom close to the E-modulus of the actual liver [40,41], as shown in Figure 11c. Finally, according to related human test references, the tumor size (including benign and malignant) of the liver cancer subjects was 0.69–15.2 cm, and mostly below 5 cm [42]. Therefore, a water ball was placed [43,44,45] to contrast with the phantom liver in the ultrasonic image in order to simulate a tumor with a diameter of 3 cm as the basis of observation and the angular correction of the RUS, as shown in Figure 11d. 

**Figure 12:**
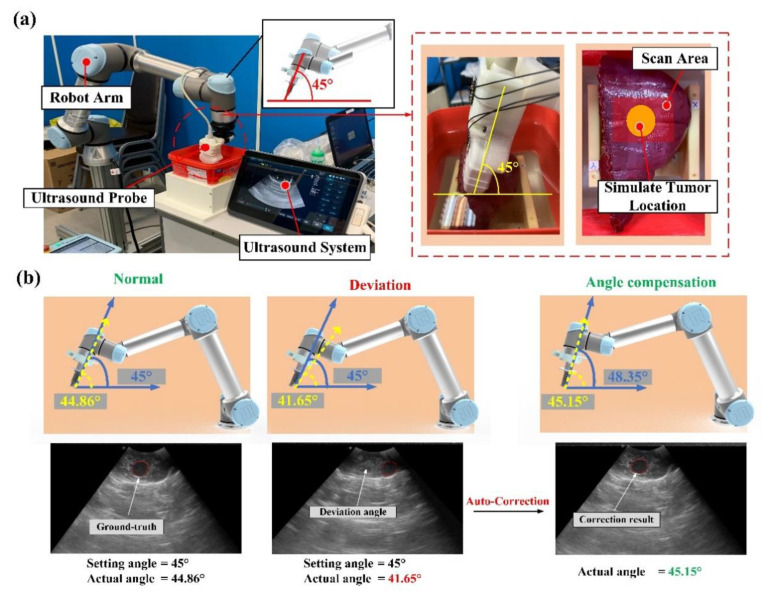
(**a**) Schematic diagram of the phantom test; (**b**) ultrasonic image test for a liver gel phantom model of the system.

First, after ensuring that the gel phantom was in a stationary state, the phantom was placed in a fixture. In order to test the method in the phantom experiment, we used a 5 mm-thick spacer to simulate the usage scenarios of the probe angles with and without deviation. In each experiment, the ultrasonic arm integrated with the ultrasonic probe fixture mechanism starts from the initial position and executes a fixed path program. The program could reach the position of tumor in the simulated liver at an angle of 45 degrees, and scan continuously for 10 seconds with a fixed force at this position. In addition to real-time image capturing, the RUS recorded and analyzed the related values. If the deviation angle was larger than the set value, the system executed the autocorrection function, and the experimental results are shown in Figure 12b. In the actual scanning of a liver phantom by the intelligent sensing device proposed in this study. When the error of angle between the ultrasonic probe and the fixture exceeded the threshold, the system could automatically correct the angle of the robot arm to get the precise ultrasound image that met the setting angle. The minimum resolution of the angle calibration was less than 2°. As shown in Figure 12b, when the system corrected the actual imaging angle, a tumor ultrasonic image with higher reproducibility could be obtained, and this was one of the advantages of the RUS in replacing manual scanning [3,4,5,6].

## 4. Discussion and Conclusions

This study successfully developed a low-cost sensing method for a RUS that could be used in ultrasonic robots through a multichannel collaborative robot arm. Moreover, angular deviation between the fixture on the robot arm and probe could be evaluated, and the probe imaging angle could be instantly corrected by the feedback mechanism, thus maintaining the good image reproducibility of the RUS. The relevant information fed back to the system and analyzed instantly could be used as reference or research data for other clinical applications of a RUS. The sensing structure proposed in this study was quite simple and could accurately identify the downforce position with an 88.2% success rate. The average error of the output force was less than 4.2%. The bilateral IMU method could detect an abnormal angle when the RUS operated effectively. The architecture proposed in this study had a low cost, high sensitivity, and high success rate, as well as good compatibility, and thus it could provide real-time abnormal angle analysis in the ultrasonic scanning process. Furthermore, the results of this study could be used in the domain of machine tactile and spatial fusion operations in the future. The information provided by this study was key to the manipulation of high DOF robot arms integrated with real-time ultrasonic robotics. In the future, the relevant results of this research could not only be applied to auxiliary system for clinical diagnosis but also be extended to related automated ultrasonic inspection systems to improve the resilience of the RUS system.

## Figures and Tables

**Table 1:** Errors of output force calculated by the regression curve equation.

Testing Force (N)	3 N	5 N	8 N	10 N
A/D value (AVG + SD)	616.6 ± 12.6	1003.8 ± 22.2	1597.3 ± 47.0	2036.1 ± 26.0
Output Force (N) (AVG + SD)	2.96 N ± 0.06 N	4.82 N ± 0.10 N	7.67 N ± 0.22 N	9.78 N ± 0.12 N
Average Error	**1.2%**	**3.7%**	**4.2%**	**2.2%**

**Table 2:** Success rate of the identification in different downforce positions.

In 28 °C of Room Temperature												
	0°	30°	60°	90°	120°	150°	180°	210°	240°	270°	300°	330°
Success Rate %(AVG ± SD)	94.8 ± 1.0	87.1 ± 1.0	83.4 ± 1.4	86.2 ± 1.4	95.9 ± 0.7	88.0 ± 1.5	84.0 ± 1.3	87.3 ± 1.6	94.6 ± 0.8	87.8 ± 1.1	82.1 ± 1.1	87.5 ± 2.1
**In 20 °C of Room Temperature**												
	0°	30°	60°	90°	120°	150°	180°	210°	240°	270°	300°	330°
Success Rate %(AVG ± SD)	94.5 ± 1.2	86.8 ± 1.4	83.5 ± 1.7	86.5 ± 1.7	94.7 ± 0.9	88.2 ± 1.4	83.9 ± 1.6	87.6 ± 1.6	94.3 ± 0.8	87.3 ± 1.7	82.4 ± 1.4	87.4 ± 2.5

There were no statistically significant differences between different temperature groups.

**Table 3:** Comparison table for the phantom test with different types of spacers.

Spacer	Setting Angle (°)	Average Measurement Angle (°)	Error (°)
Type A (*d* = 2.5 mm)	45°	46.3°	1.3°
	90°	91.2°	1.2°
	135°	136.5°	1.5°
Type B (*d* = 5.0 mm)	45°	47.7°	2.7°
	90°	93.1°	3.1°
	135°	137.5°	2.5°
Type C (*d* = 10.0 mm)	45°	49.8°	4.8°
	90°	95.9°	5.9°
	135°	139.9°	4.9°

*d* means thickness of the spacer.

## Data Availability

Data sharing is not applicable to this article.

## References

[1] Nakadate R., Solis J., Takanishi A., Sugawara M., Niki K., Minagawa E. (2010). Development of the ultrasound probe holding robot WTA-1RII and an automated scanning algorithm based on ultrasound image feedback. ROMANSY 18 Robot Design, Dynamics and Control.

[2] Harrison G., Harris A. (2015). Work-related musculoskeletal disorders in ultrasound: Can you reduce risk?. Ultrasound.

[3] Freschi C., Ferrari V., Melfi F., Ferrari M., Mosca F., Cuschieri A. (2013). Technical review of the da Vinci surgical telerobot arms. Int. J. Med. Robot. Comput. Assist. Surg..

[4] Sutherland G.R., Wolfsberger S., Lama S., Zarei-nia K. (2013). The evolution of neuroArm. Neurosurgery.

[5] Samei G., Tsang K., Kesch C., Lobo J., Hor S., Mohareri O., Chang S., Goldenberg L., Black P.C., Salcudean S. (2020). A partial augmented reality system with live ultrasound and registered preoperative MRI for guiding robot-assisted radical prostatectomy. Med. Image Anal..

[6] Du Y.C., Shih J.B., Wu M.J., Chiou C.Y. (2018). Development of an AVF Stenosis Assessment Tool for Hemodialysis Patients Using Robotic Ultrasound System. Micromachines.

[7] Oleari E., Leporini A., Trojaniello D., Sanna A., Capitanio U., Dehó F., Larcher A., Montorsi F., Salonia A., Muradore R. Enhancing surgical process modeling for artificial intelligence development in robotics: The saras case study for minimally invasive procedures. Proceedings of the 2019 IEEE 13th International Symposium on Medical Information and Communication Technology (ISMICT).

[8] Li Z., Kang Y., Xiao Z., Song W. (2016). Human–robot coordination control of robotic exoskeletons by skill transfers. IEEE Trans. Ind. Electron..

[9] Zinchenko K., Wu C.Y., Song K.T. (2016). A study on speech recognition control for a surgical robot. IEEE Trans. Ind. Inform..

[10] Li G., Su H., Cole G.A., Shang W., Harrington K., Camilo A., Pilitsis J.G., Fischer G.S. (2014). Robotic system for MRI-guided stereotactic neurosurgery. IEEE Trans. Biomed. Eng..

[11] Swerdlow D.R., Cleary K., Wilson E., Azizi-Koutenaei B., Monfaredi R. (2017). Robotic arm–assisted sonography: Review of technical developments and potential clinical applications. Am. J. Roentgenol..

[12] Mustafa A.S.B., Ishii T., Matsunaga Y., Nakadate R., Ishii H., Ogawa K., Saito A., Sugawara M., Niki K., Takanishi A. Development of robotic system for autonomous liver screening using ultrasound scanning device. Proceedings of the 2013 IEEE international conference on robotics and biomimetics (ROBIO).

[13] Huang Q., Lan J. (2019). Remote control of a robotic prosthesis arm with six-degree-of-freedom for ultrasonic scanning and three-dimensional imaging. Biomed. Signal Process. Control.

[14] Chen S., Wang F., Lin Y., Shi Q., Wang Y. (2021). Ultrasound-guided needle insertion robotic system for percutaneous puncture. Int. J. Comput. Assist. Radiol. Surg..

[15] Zhang W.J., Van Luttervelt C.A. (2011). Toward a resilient manufacturing system. CIRP Ann..

[16] Adams S.J., Burbridge B., Obaid H., Stoneham G., Babyn P., Mendez I. (2020). Telerobotic Sonography for Remote Diagnostic Imaging: Narrative Review of Current Developments and Clinical Applications. J. Ultrasound Med..

[17] Ye R., Zhou X., Shao F., Xiong L., Hong J., Huang H., Tong W., Wang J., Chen S., Chen L. (2020). Feasibility of a 5G-Based Robot-Assisted Remote Ultrasound System for Cardiopulmonary Assessment of Patients with Coronavirus Disease 2019. Chest.

[18] Tsumura R., Hardin J.W., Bimbraw K., Odusanya O.S., Zheng Y., Hill J.C., Hoffmann B., Soboyejo W., Zhang H.K. (2020). Tele-operative Robotic Lung Ultrasound Scanning Platform for Triage of COVID-19 Patients. arXiv.

[19] Porpiglia F., Checcucci E., Amparore D., Piramide F., Volpi G., Granato S., Verri P., Manfredi M., Bellin A., Mottrie A. (2020). Three-dimensional augmented reality robot-assisted partial nephrectomy in case of complex tumours (PADUA ≥ 10): A new intraoperative tool overcoming the ultrasound guidance. Eur. Urol..

[20] Fontanelli G.A., Buonocore L.R., Ficuciello F., Villani L., Siciliano B. A novel force sensing integrated into the trocar for minimally invasive robotic surgery. Proceedings of the 2017 IEEE/RSJ International Conference on Intelligent Robots and Systems (IROS).

[21] Yu L., Yan Y., Yu X., Xia Y. (2018). Design and realization of forceps with 3-D force sensing capability for robot-assisted surgical system. IEEE Sens. J..

[22] Li X., Kesavadas T. Surgical robot with environment reconstruction and force feedback. Proceedings of the 2018 40th Annual International Conference of the IEEE Engineering in Medicine and Biology Society (EMBC).

[23] McInroe B.W., Chen C.L., Goldberg K.Y., Bajcsy R., Fearing R.S. Towards a soft fingertip with integrated sensing and actuation. Proceedings of the 2018 IEEE/RSJ International Conference on Intelligent Robots and Systems (IROS).

[24] Cramphorn L., Lloyd J., Lepora N.F. Voronoi features for tactile sensing: Direct inference of pressure, shear, and contact locations. Proceedings of the 2018 IEEE International Conference on Robotics and Automation (ICRA).

[25] Zhang Y., Zhang G., Du Y., Wang M.Y. VTacArm. A Vision-based Tactile Sensing Augmented Robotic Arm with Application to Human-robot Interaction. Proceedings of the 2020 IEEE 16th International Conference on Automation Science and Engineering (CASE).

[26] Alakhawand N., Frier W., Freud K.M., Georgiou O., Lepora N.F. (2020). Sensing Ultrasonic Mid-Air Haptics with a Biomimetic Tactile Fingertip. International Conference on Human Haptic Sensing and Touch Enabled Computer Applications.

[27] Toyama S., Tanaka Y., Shirogane S., Nakamura T., Umino T., Uehara R., Okamoto T., Igarashi H. (2017). Development of wearable sheet-type shear force sensor and measurement system that is insusceptible to temperature and pressure. Sensors.

[28] Uneo Pressure Sensor, GD10-20N Spec Sheet. http://www.uneotech.com/uneo/online-store/96/gs0001-4-uneo.html.

[29] Xiloyannis M., Galli L., Chiaradia D., Frisoli A., Braghin F., Masia L. (2018). A soft tendon-driven robotic glove: Preliminary evaluation. International Conference on Neurorehabilitation.

[30] Mansfield S., Rangarajan S., Obraczka K., Lee H., Young D., Roy S. Objective Pressure Injury Risk Assessment Using A Wearable Pressure Sensor. Proceedings of the 2019 IEEE International Conference on Bioinformatics and Biomedicine (BIBM).

[31] Octopart, MPU-6050. https://octopart.com/mpu-6050-invensense-19505926?gclid=CjwKCAiAgJWABhArEiwAmNVTB89XRStZacmvsS9k_uGfsrPDFcUTHsO5KUI4UV_wCtCSt8Bvg8kBYRoCo6IQAvD_BwE.

[32] Ding Z.Q., Luo Z.Q., Causo A., Chen I.M., Yue K.X., Yeo S.H., Ling K.V. (2013). Inertia sensor-based guidance system for upperlimb posture correction. Med. Eng. Phys..

[33] Wen T., Wang C., Zhang Y., Zhou S. (2020). A Novel Ultrasound Probe Spatial Calibration Method Using a Combined Phantom and Stylus. Ultrasound Med. Biol..

[34] Poon T.C., Rohling R.N. (2005). Comparison of calibration methods for spatial tracking of a 3-D ultrasound probe. Ultrasound Med. Biol..

[35] Du Y.C., Shih C.B., Fan S.C., Lin H.T., Chen P.J. (2018). An IMU-compensated skeletal tracking system using Kinect for the upper limb. Microsyst. Technol..

[36] Chen P.J., Du Y.C., Shih C.B., Yang L.C., Lin H.T., Fan S.C. Development of an upper limb rehabilitation system using inertial movement units and kinect device. Proceedings of the 2016 International Conference on Advanced Materials for Science and Engineering (ICAMSE).

[37] Gillies D.J., Bax J., Barker K., Gardi L., Tessier D., Kakani N., Fenster A. (2019). Mechanically assisted 3D ultrasound with geometrically variable imaging for minimally invasive focal liver tumor therapy. Medical Imaging 2019: Image-Guided Procedures, Robotic Interventions, and Modeling.

[38] Hu J., Zhou Z.Y., Ran H.L., Yuan X.C., Zeng X., Zhang Z.Y. (2020). Diagnosis of liver tumors by multimodal ultrasound imaging. Medicine.

[39] Ahmad M.S., Suardi N., Shukri A., Ab Razak N.N.A.N., Oglat A.A., Makhamrah O., Mohammad H. (2020). Dynamic Hepatocellular Carcinoma Model Within a Liver Phantom for Multimodality Imaging. Eur. J. Radiol. Open.

[40] Harbin W.P., Robert N.J., Ferrucci J.T. (1980). Diagnosis of cirrhosis based on regional changes in hepatic morphology: A radiological and pathological analysis. Radiology.

[41] Ilione T., Ohagwu C.C., Ogolodom M.P. (2019). Computed Tomography evaluation of the Caudate-to-Right Lobe ratio in Patients with Liver Cirrhosis and Subjects with Normal Liver in Benin City, Edo State, Nigeria. Health Sci. J..

[42] da Silva N.P.B., Hornung M., Beyer L.P., Hackl C., Brunner S., Schlitt H.J., Wiggermann P., Jung E.M. (2019). Intraoperative shear wave elastography vs. contrast-enhanced ultrasound for the characterization and differentiation of focal liver lesions to optimize liver tumor surgery. Ultraschall Med. Eur. J. Ultrasound.

[43] Gerling G.J., Thomas G.W. (2005). Augmented, pulsating tactile feedback facilitates simulator training of clinical breast examinations. Hum. Factors.

[44] Jeon S., Choi S., Harders M. (2011). Rendering virtual tumors in real tissue mock-ups using haptic augmented reality. IEEE Trans. Haptics.

[45] Kaneko H., Sano H., Hasegawa Y., Tamura H., Suzuki S.S. (2017). Effects of forced movements on learning: Findings from a choice reaction time task in rats. Learn. Behav..

